# Realizing quasi-monochromatic switchable thermal emission from electro-optically induced topological phase transitions

**DOI:** 10.1038/s41598-022-11410-6

**Published:** 2022-05-05

**Authors:** Nitish Kumar Gupta, Sapireddy Srinivasu, Anjani Kumar Tiwari, Harshawardhan Wanare, S. Anantha Ramakrishna

**Affiliations:** 1 Centre for Lasers & Photonics, Indian Institute of Technology Kanpur, Kanpur, 208016 India; 2 Department of Physics, Indian Institute of Technology Roorkee, Roorkee, 247667 India; 3 Department of Physics, Indian Institute of Technology Kanpur, Kanpur, 208016 India; 4 CSIR-Central Scientific Instruments Organisation, Chandigarh, 160030 India

**Keywords:** Applied optics, Optical materials and structures, Micro-optics, Sub-wavelength optics

## Abstract

Explorations into the photonic analogs of topological materials have garnered significant research interest due to their application potential. Particularly in planar systems, the prospects of engendering extinguishable topological states can have wide-ranging implications. With an objective of employing these concepts for thermal emission engineering, here, we design and numerically investigate a quasi-monochromatic highly directional mid-infrared source elicited from inversion symmetry-protected topological interface states. Notably, by relying on the architecture of electro-optic effect-induced topological phase transitions, we introduce the possibility of ultrafast switching of thermal radiation. These reversible phase transitions, being free from carrier transport are inherently fast and evoke thermal emission modulation with a modulation depth upto 0.99. Specifically, our platform exhibits a near-perfect extinguishable spectral emission peak at $4~\mu$m with a quality factor of over 18500, displaying negligible parasitic emissions. Furthermore, the optimized interface state manifests itself for only one of the polarization modes, resulting in polarized emission under resonance conditions. To establish a methodical approach to parameter optimization, we also model our platform as a leaky mode resonator using the framework of temporal coupled-mode theory. We believe, our findings can provide a way forward in establishing complete control over the optical characteristics of the infrared thermal emitters.

## Introduction

Attributable to its statistical nature, the thermal emission is considered to be lacking the features of selectivity and hence almost exclusively associated with characteristics such as broadband spectrum, unpolarized emission, and a quasi-isotropic angular emittance^1^. Therefore, the prospects of exhibiting control over thermal radiation are of widespread fundamental significance, apart from being crucial to the design and operation of devices ranging from micron-scale chips to astronomical size systems. Inter alia, the fruition of thermal emission with ultranarrow bandwidths has recently garnered significant research interest owing to its direct utility in applications ranging from - high figure-of-merit infrared (IR) sensing, IR spectroscopy and fingerprinting, thermophotovoltaics, photon mediated thermal logic, IR labeling, anti-counterfeiting, and in countermeasures development against IR homing projectiles^2–6^. The development of techno-commercially plausible customized thermal sources, particularly in the mid-IR domain, however, has its challenges. Owing to the fact that the spontaneous emission scales at cubic power of frequency, an IR analog of LED turns out to be highly inefficient^7^. Although state-of-the-art solutions like Quantum Cascade Lasers can surpass most of the spectral purity requirements, the intersubband transitions based operation requires stacking up of many hundreds of quantum wells and barrier layers^8^. Motivated by these viability concerns and to develop application-specific minimalistic solutions, many researchers have revisited the problem of optimizing the radiant emittance per unit area from an object (defined in terms of the Planck’s law of spectral radiance)^9^ and noticed that although the temperature management is one obvious way to control the thermal emission; however, the theory also suggests a trickier but more rewarding alternative of emissivity engineering^10–20^. This realization has brought the nano, and microphotonic structures to the forefront, which can artificially alter the absorptivity/emissivity using structure-derived electromagnetic resonances. Such designs provide an austere yet amply suited template to control the light-matter interaction in a way that near-perfect spectrally selective, efficient, and highly directional absorption can be materialized. As established by Kirchhoff’s law, under thermodynamic equilibrium, the reciprocity can be factored in and the same concepts also provide an equivalent assessment of emission. Over the years, however, it has been observed that many of these proposals also resort to complicated lithography techniques^1, 21^, thereby raising concerns of structural stability at high temperatures apart from a limited commercial acceptance. Additionally, significant background and multiple resonances lead to high noise levels and wasteful parasitic emissions, affecting the conversion efficiencies and the achievable signal-to-noise ratios. Furthermore, these proposals cater only to the static spectral selectivity requirements. High-speed switching and modulation have been perennially considered incompatible with thermal emission due to inherently slow dynamics of heat transport, even for devices with small thermal inertia. This had limited the modulation speeds up to 100 Hz^7, 22^; only of-late the proposals have been put forward to achieve MHz switching^23–25^. However, a thermal emission modulation scheme free from carrier transport and complying with thermodynamic equilibrium can offer orders of magnitude faster speeds, motivating us to look for atypical replacements.

On a different note, the application of topology, a concept of modern mathematics, to the condensed matter physics led to the discovery of neoteric and exciting paradigms such as topological phases of matter and effective magnetic fields^26^. Particularly, in Fermionic systems, the inception of band topology dates back to the seminal work by Thouless et al.^27^, which has been gradually extended to classical electromagnetic wave systems^28^. The keystone of the formalism lies in conception that the momentum space global properties of a topologically non-trivial bulk bandstructure can be encapsulated in quantized topological invariants^26^, and by combining structures of different topological orders, unconventional interface or edge states can be manifested with detailed control. These topological interface states (TIS) provide a doorway to additional degrees of design freedom, consequently garnered enormous research interest and facilitated the dawn of topological photonics^26, 28–35^.

Our present work concentrates on employing the principles of topological photonics to conceptualize topological phase transitions in a spatial inversion symmetric (SIS) multilayer configuration, which being relieved from inertial aspects, can lead to the ultrafast switching of TIS. The dynamic topological phase transition in our structure is induced by the repetitive system evolution across a Brillouin Zone (BZ) edge bandgap closing point using the electro-optic (EO) effect of anisotropic LiNbO$_{3}$ films. The resulting symmetry-protected TIS is then integrated with a localized loss of atomically thin monolayer graphene, leading to a monochromatic thermal emitter with temporally agile modulation capabilities. Notably, the movement across the topological phase transition point assigns a new degree of design freedom to the realized interface state where its very existence can be controlled by weak modulations in the refractive index. The platform is designed to operate in a technologically important atmospheric high transmission window at mid-IR ($3-5~\mu$m). Specifically, the optimizations have been performed to synthesize the emitter at the middle of a photonic bandgap at $4~\mu$m; this provides high selectivity and rules out the possibility of parasitic emissions leading to high conversion efficiencies. Furthermore, by circumventing the patterning requirements, our platform not only ensures structural stability at high operating temperatures but also provides means for mass production using standard deposition techniques.

## A routine for bandstructure engineering

The platform under consideration here consists of a binary one-dimensional photonic crystal (1D PhC) constituting from spatial inversion symmetric unit cells. In such a stratified medium, the Bloch waves $E_{n,K}^{z}(x)=u_{n,K}(x) e^{-iKx}$ define the eigenmodes of the system, with $u_{n,K}(x)$ being the periodic part of the Bloch solution, matching the periodicity of the structure $\Lambda$, i.e. $u_{n,K}(x+\Lambda )=u_{n,K}(x)$. The bandstructure calculations for this system can be performed using the dispersion relation^36^

1 $$\begin{aligned} \cos (K\Lambda )= \cos (k_{a}d_{a})\cos (k_{b}d_{b})-\frac{1}{2}\left( \frac{Z_{a}}{Z_{b}} + \frac{Z_{b}}{Z_{a}}\right) \sin (k_{a}d_{a})\sin (k_{b}d_{b}) \end{aligned}$$

**Figure 1:**
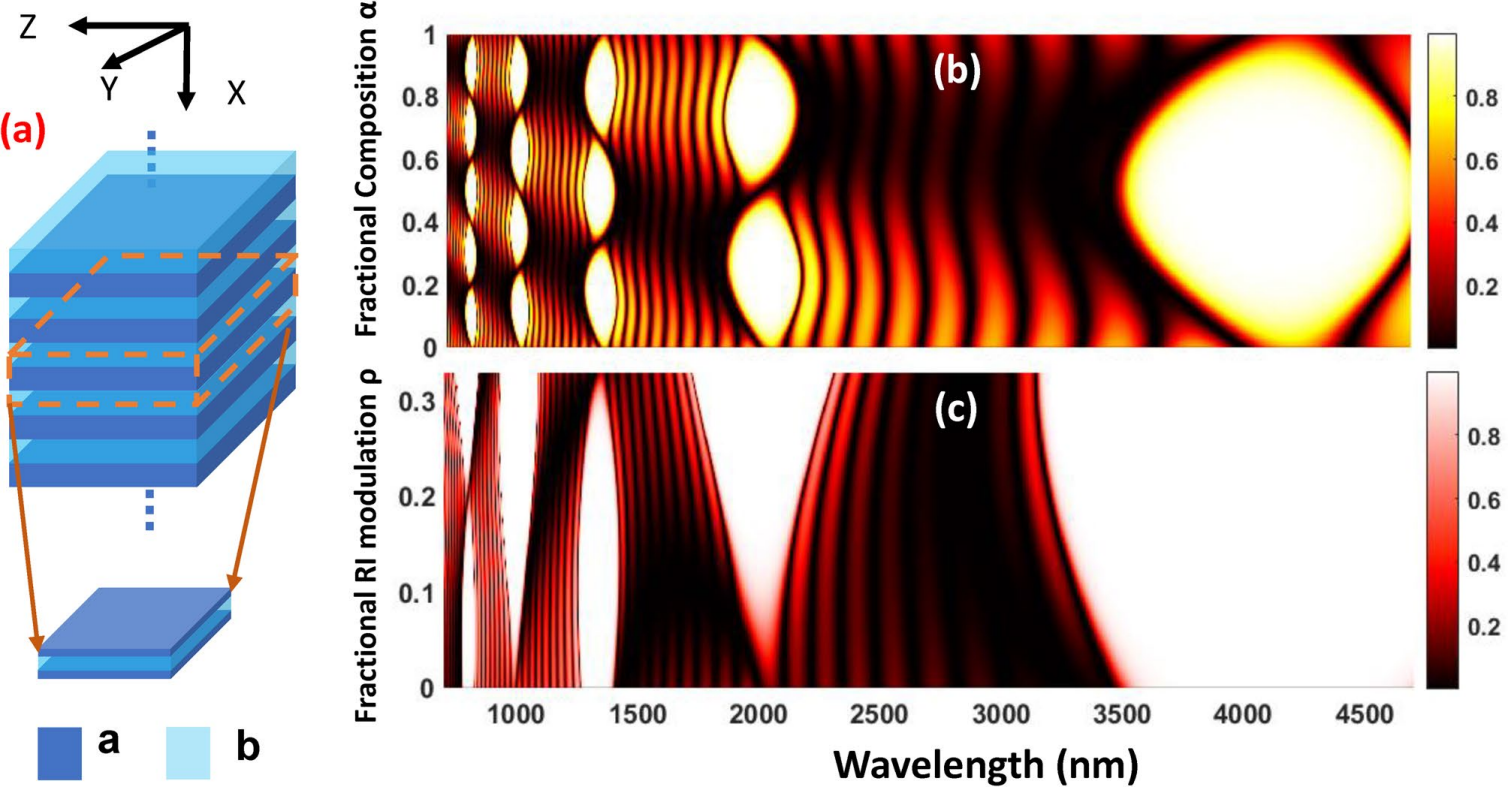
(**a**) Schematic for a 1D PhC with inversion sysmmetric unit cells (dashed line in schematic); (**b**) parametric behaviour of PhC reflectance with the unit-cell composition fraction $\alpha$; (c) parametric behaviour of PhC reflectance with the fractional RI modulation parameter $\rho$.

where *K* is the Bloch momentum; ${Z_{i}}=(\mu _{i}/~\epsilon _{i})$ is the impedance and $k_i$ is the wavenumber in the $i^{\text {th}}$ medium ($i=a,b$); $\Lambda$ is the lattice constant ($\Lambda =d_{a}$+$d_{b}$). The absolute value of the right-hand side (RHS) of the Eq (1) assigns the frequency regions corresponding to the bands and bandgaps. When it is smaller than or equal to 1, the equation gets satisfied for a real value of Bloch momentum *K*, and the corresponding solutions are the propagating modes defining a passband. On the contrary, if the absolute value of the RHS is greater than 1, only imaginary values of *K* can satisfy the equation leading to exponentially decaying states denoting a bandgap. It has been explained earlier^37^ that the maxima and minima of RHS define the midgap frequencies, whose location can be approximately ascertained from the expression $\omega _{m}= m\frac{\pi c}{n_a d_a +n_b d_b}$ (with m being an integer and $n_a$, $n_b$ are the refractive indices corresponding to the medium ‘a’ and ‘b’). Therefore, although the bandstructure itself is sensitive to the unit cell composition, the positions of midgap frequencies will remain oblivious to it, determined solely by the total optical thickness ($n_a d_a +n_b d_b$). This provides an opportunity for calibrated manipulation of the bandstructure and a venue to study bandgap/band transitions. To fathom the resulting implications, we proceed in a systematic manner by first quantifying the fractional composition of the unit-cell (depicted in Fig. 1a) and then using it to study the parametric performance of a representative PhC. Accordingly, we define the fractional weightage of material ‘a’ in the unit cell as $\alpha =\frac{n_a d_a}{n_a d_a +n_b d_b}$ and parametrize the individual layer thicknesses in terms of it. Thereafter keeping the optical thickness constant, we scan the entire parametric space of $\alpha$ to obtain the reflectance performance of a PhC consisting of 10 periods with $n_a=2.0$ and $n_b=3.0$ (the design wavelength corresponding to $1^{st}$ order bandgap is taken to be $4~\mu {m}$). These calculations are performed using the standard transfer matrix method (TMM), and the results are plotted in Fig. 1b, highlighting the following attributes: (1) from the topological standpoint, the first order bandgap is trivial as it entails no prospects for band inversion; only from $2^{nd}$ bandgap onwards we see the occurrence of probable band inversion sites. (2) As we move to higher-order bandgaps, the fractional change required in the unit-cell composition for an open-close-open transition comes down significantly.

Moving on, it is noteworthy to mention that if we are interested in realizing a topological interface state only, this route of thickness parametrization turns out to be more conducive for devising a fabricable design and will be implemented elsewhere. However, in this work, since we aim for temporal modulation of TIS, it warrants dynamic manipulation of topological invariants of the system. Therefore, external means of dynamic bandstructure tuning must be called in. To this objective, we resort to the EO effect-induced perturbative changes in the refractive index (RI). For exploring the sites of band inversion, we look for refractive index parametrization in a representative PhC. Such a parametrization, however, is not as forthright and requires more careful investigation as a change in the RI contrast would have wide-ranging impacts on bandstructure. Nevertheless, we begin by defining a new parameter $\rho =\delta {n_i}/{n_i}$ which quantifies the fractional change in RIs of PhC constituents around their base values (keeping optical thickness constant) and obtain the reflectance characteristics of a 10 period PhC as done previously (with base RIs $n_a=2$, $n_b=3$ and fixed thicknesses of $d_a=0.5\mu {m}$, $d_b= 0.33\mu {m}$ corresponding to $\alpha =0.5$). We also want to point out that the configuration adopted here would lead to an increase in RI contrast with an increasing value of $\rho$ (which is more aligned with the case of LiNbO3-Si platform to be used later on). Notice here that the parametric space of $\rho$ is highly restricted not only on account of the practical limitations but also keeping in mind that it would make little sense to talk of $\rho =1$. The calculation results are plotted in Fig. 1c, sufficiently highlighting the fact that the small RI alterations possible with the EO effect will always be insufficient in realizing the band inversion for lower-order bandgaps.

The ongoing analysis, therefore, encourages us to safely assume that in the present work, we have to invariably work with higher-order bandgaps. Equipped with these insights, in the next section, we realize a TIS modulator around the $5^{th}$ order bandgap for a concatenated PhC structure.

## Results: electro-optically switchable topological phase transitions

**Figure 2:**
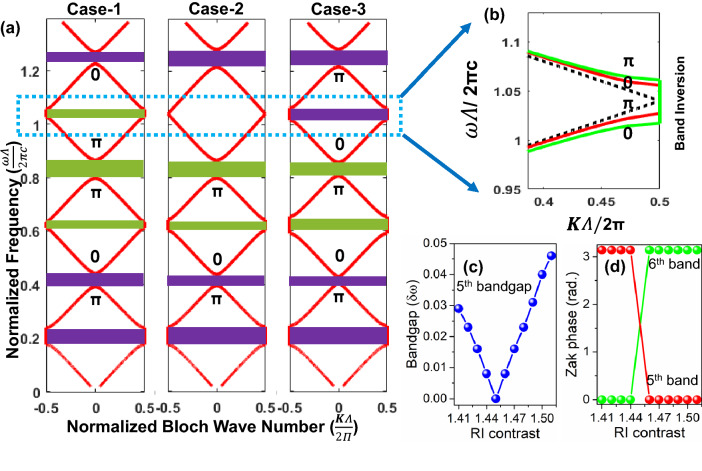
(**a**) Folded bandstructure of a periodic 1D PhC with inversion symmetric unit cell for three topologically distinct cases; (**b**) Topological phase transition around the $5^{\text {th}}$ order bandgap; (**c**) Bandgap evolution with small changes in the PhC RI contrast, and (**d**) corresponding transitions in the topological invariant.

Our objective is to first realize a PhC platform admitting an EO effect-induced manipulation in its bandstructure (and a possible band inversion). We commence the design with material considerations: on account of the proven compatibility of LiNbO$_{3}$ with the silicon-based platforms, the constituent materials have been selected to be Si and LiNbO$_{3}$ having a RI of 3.45 and 2.04 respectively in mid-IR^38, 39^. The electro-optically induced change in LiNbO$_{3}$ RI can be ascertained by the following relation:

2 $$\begin{aligned} \Delta \left( \frac{1}{n^{2}}\right) _{ij} =\sum _{k}r_{ijk}E_{k}, \end{aligned}$$

where $\Delta (\frac{1}{n^{2}})_{ij}$ is the second-rank tensor of the change in relative permittivity, $r_{ijk}$ is a third-rank linear EO coefficient tensor, $E_{k}$ is electric field component along k; i,j,k correspond to x,y, and z coordinate axes respectively. Among all the EO coefficients $r_{ijk}$, the $r_{33}$ is the largest linear EO (Pockel’s) coefficient (see Supplementary Information). Therefore, in this work, we choose a configuration such that $r_{33}$ is employed. Moving forward, to map out the realistic range of RI modulation facilitated by the EO effect, we have resorted to some of the previously published reports^40–42^ and the design considerations mentioned in them, which provide us a span of 2.04 to 1.94. A reduction in RI takes place with the applied voltage on account of the negative uniaxial nature of LiNbO$_{3}$. With the required materials in place, we now embark on an expedition to capture an EO effect-induced topological phase transition in the momentum space and try to fathom how it can promulgate an extinguishable TIS. Equipped with the previously gained insights, we search for an open-close-open transition in the vicinity of $5^{th}$ order bandgap and successfully obtain one. The results of bandstructure calculations as per Eq (1), within the mentioned span of available RIs, for three representative scenarios have been plotted in Fig. 2a: Case-1 marks the reference case under no external field with Si and LiNbO$_{3}$ thicknesses of 3.08 and $1.07~\mu$m; Case-2 corresponds to a moderate application of field and the associated reduction in LiNbO$_{3}$ RI to 2.00; while Case-3 denotes a further reduction to a value of 1.94. Noteworthy to mention that in order to illustrate the concepts, thicknesses in Case-2 and 3 have been adjusted for ensuring the invariance of band centers. Thereafter, the topological character of the bulk bands has been ascertained by the extraction of geometric Zak phases^37^ calculated using Eq (3) and denoted alongside the bands in bold black letters.

3 $$\begin{aligned} \theta ^{Zak}_{n}=\int ^{\pi /\Lambda }_{-\pi /\Lambda }\left( i\int _{unit~cell}dz~\epsilon (z)u^{*}_{n,K}(z) \frac{ \partial }{ \partial K}u_{n,K}(z)\right) dK \end{aligned}$$

where $\theta ^{zak}_{n}$ is the Zak phase associated with $n^{\text {th}}$ band; the quantity in the parenthesis is the Berry connection (or Zak connection) of the $n^{\text {th}}$ band ($\varvec{\mathscr {A}}^{Zak}_{n}$); $\epsilon (z)$ is spatial permittivity function and $u_{n,K}(z)$ is the Bloch eigenfunction in the $n^{\text {th}}$ band, which is obtained using the established eigenvalue formulation followed in stratified media^36^. From the bandstructure evolution presented in Fig. 2a and b and the corresponding Zak phase calculations, we notice that the open-close-reopen transition at the $5^{\text {th}}$ order bandgap indeed corresponds to a band inversion and an associated reversal of topological invariants (Zak phases) for the neighboring bands. This proclaims a transition in momentum space topology of bulk bands with a change in LiNbO$_{3}$ RI . The evolution of bandgap width and Zak phases have been specifically captured in Fig. 2c and d with RI contrast (defined as difference between the RIs of Si and LiNbO3). According to the bulk-interface correspondence, ramifications of such a transition should be traceable as a sign reversal in the surface impedance of the associated bandgaps^37^, which can be ascertained by following the evolution of Zak phases of all the bulk bands below it, resulting in

4 $$\begin{aligned} sgn[\xi ^{(n)}]=(-1)^{n}(-1)^{l}exp\left( i\sum ^{n-1}_{m=0}\theta ^{Zak}_{m}\right) \end{aligned}$$

where $\xi ^{(n)}$ is defined as $i\xi ^{(n)}=\frac{Z^{(n)}}{Z_{0}}$; *l* is the number of crossings under the $n^{\text {th}}$ bandgap. Calculations as per Eq (4) confirm this proposition which has been explicitly represented in Fig. 2a by color-coding the bandgaps. In this formalism, we can see that the surface impedance of the $5^{\text {th}}$ order bandgap reverses its sign (color) during the transition from Case-1 to 3, engendering the possibility of a topological interface state.

**Figure 3:**
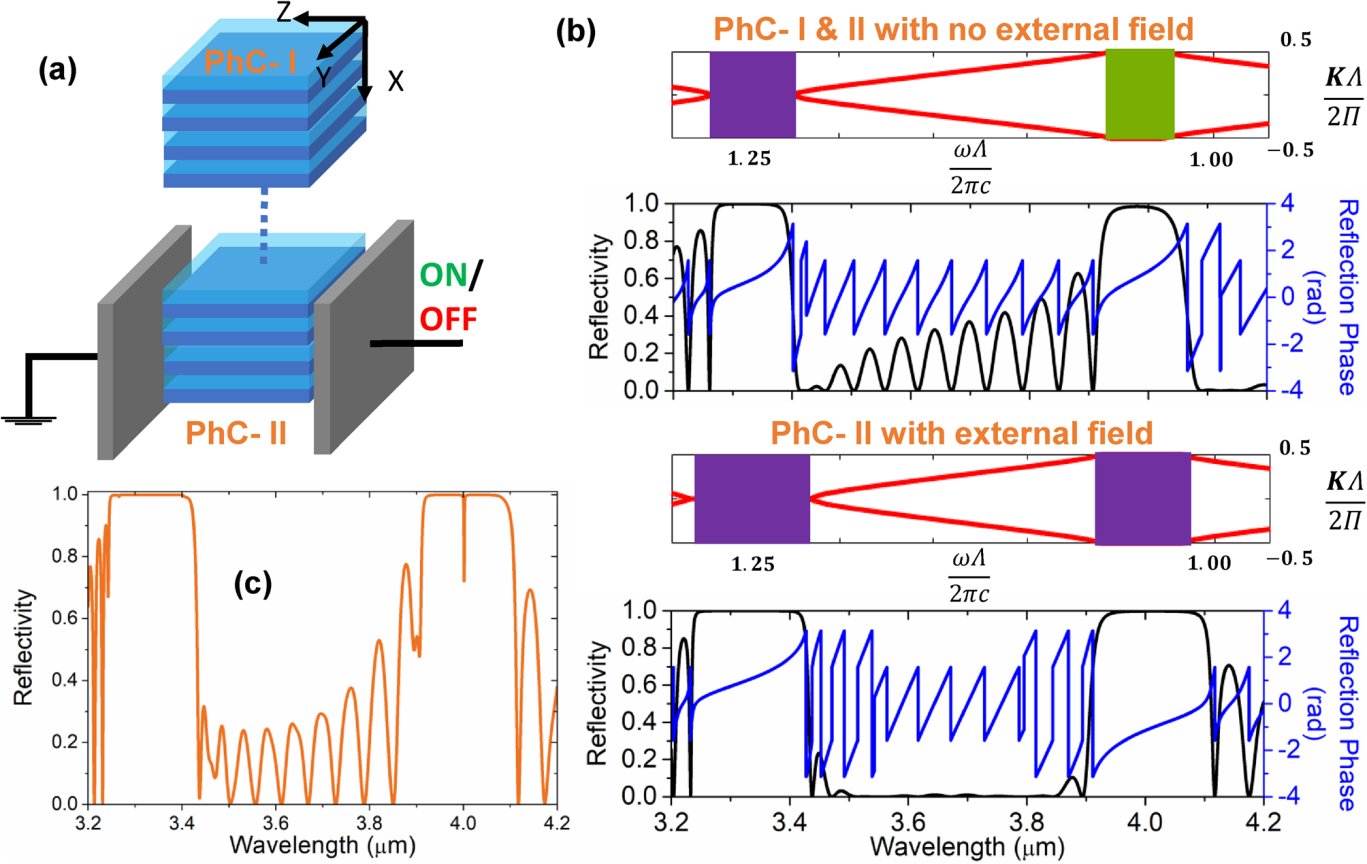
(**a**) Schematic for the TIS modulator; (**b**) electro-optically induced phase transition: bandstructure and scattering characteristics of the constitutive PhCs with and without external electric field; (**c**) manifestation of an extinguishable TIS in the integrated assembly.

To witness the manifestation of such a TIS, we create a defect-free assembly of PhC-I and PhC-II (in the sense that there is no defect layer incorporation and no break in periodicity) having periods N$_{1} = \text {N}_{2} = 10$, and obtain the concatenated structure of Fig. 3a, which will be termed as a TIS modulator. While the PhC-I always retains the character of Case-1, the PhC-II can make a phase transition from Case-1 to Case-3, depending upon the externally applied field and the resulting perturbative change in RI. During this transition, the surface impedance sign reversal translates to reflection phase reversal as plotted in Fig. 3b. It brings us to a dynamic conjugate impedance matching condition: when the modulator is in ’ON’ state, a TIS emerges at $4~\mu$m (Fig. 3c) on account of mismatch in topological invariants of the two constituent PhCs. In the ’OFF’ state, however, the TIS gets extinguished (see Fig. 1 in Supplementary Information) as the topological invariants of PhC-I and PhC-II become equal. For vindicating the interface state nature of TIS, the electric field magnitude profile of TIS at the resonant wavelength of $4~\mu {m}$ has also been provided in Supplementary Information.

**Figure 4:**
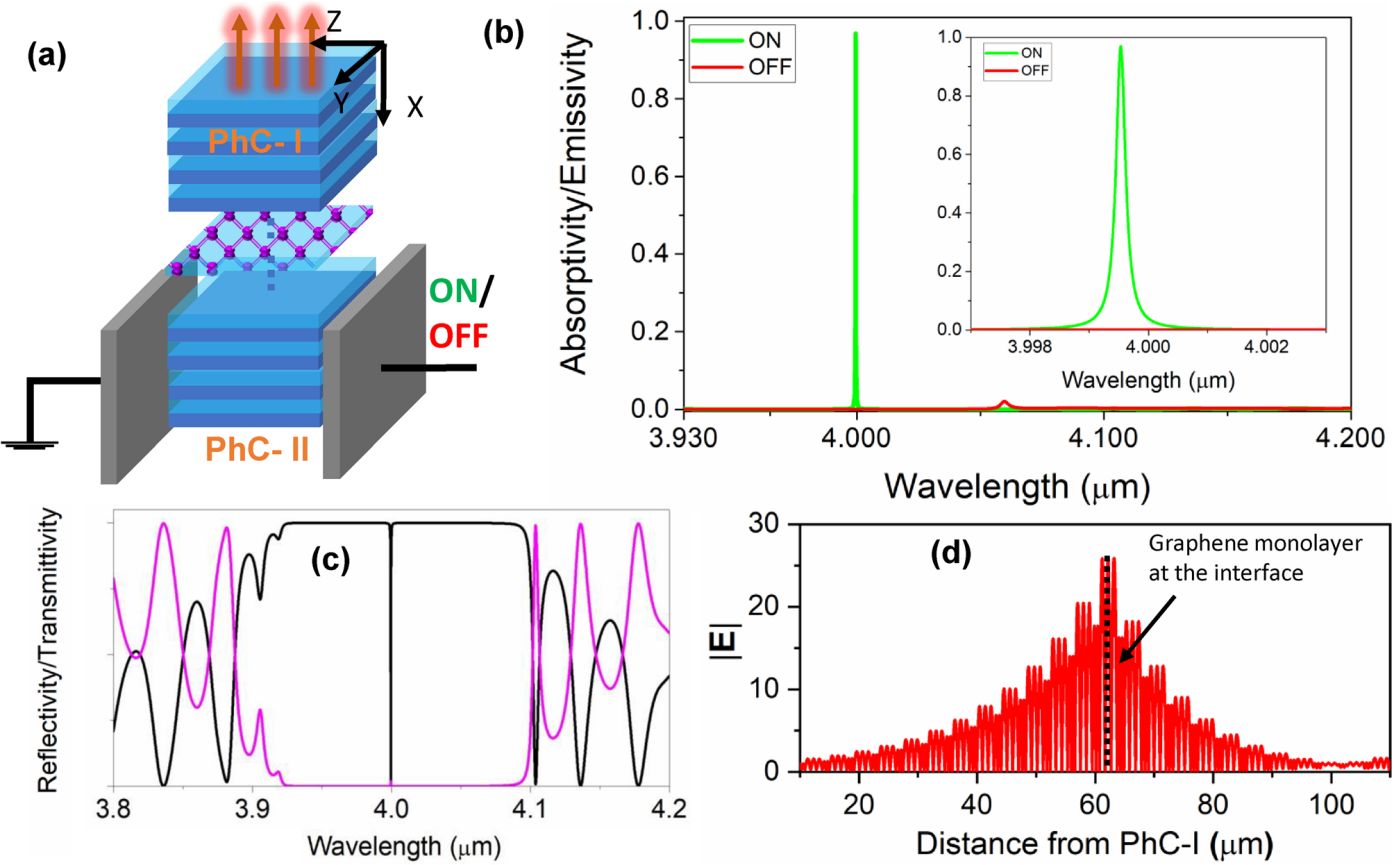
(**a**) Schematic for the engineered thermal emitter employing monolayer graphene; (**b**) switching characteristics of thermal emission: calculated spectral emissivity characteristics for two logic states; (**c**) reflectivity/ transmissivity spectra under resonance condition (ON state), (**d**) electric field magnitude profile at peak emission wavelength of $4~\mu {m}$.

Before moving any further, we also succinctly explain the polarization-related modalities of the obtained TIS. In the concatenated PhC structure of Fig. 3a, we have chosen the optic axis and the applied voltage to be along the z-axis (with light propagating along the x-axis). As noted before, in such a configuration, the Pockel’s coefficient $r_{33}$ comes into the picture for z-polarized light, and the corresponding maximum change of the RI in $n_{z}$ is given as

5 $$\begin{aligned} \delta n_{z}=-\frac{n_{e}^{3}}{2}r_{33}E_{z}, \end{aligned}$$

where $n_{e}$ is extraordinary RI. The applied electric field instigates a RI change in the $n_{y}$ as well, which can be determined as $\delta n_{y}=-\frac{n_{o}^{3}}{2}r_{13}E_{z}$ where $n_{o}=2.11$ is ordinary RI at $\lambda =4~\mu m$. The EO coefficient $r_{13}$ employed here is about five times smaller in value than $r_{33}$ in the wavelength region of interest; in consequence, the RI change $\delta n_{y}$ would be sufficiently smaller than $\delta n_{z}$. This disparity will ensure the occurrence of topological phase transition for only one of the polarization modes (z-polarization) and bring polarization discrimination into the structure.

With the architecture of a switchable TIS in place, we introduce localized ohmic loss to the structure in the form of a monolayer Graphene^43^ (Fig. 4a), the optical properties of which have been modeled using the Kubo formalism^44^, accounting for both, the interband and intraband contributions to the graphene conductivity (see Supplementary Information). The arrangement is then optimized for N$_{1}$ and N$_{2}$ to realize near-perfect absorption/emission with results plotted in Fig. 4b for N$_{1} = 15$ and N$_{2} = 16$. Manifestation of TIS in ON-state evinces out an ultra-narrow spectral resonance with an emissivity peak of 0.97 at $4~\mu$m, leading to coherent infrared emission. The corresponding linewidth and associated quality factor come out to be $0.0002~\mu$m and 18600. The OFF-state response is also plotted in red, demonstrating a high modulation depth (0.99) and extinction ratio ($-19.4$ dB) for our platform. The associated scattering performance is plotted in Fig. 4c with black curve denoting a near-perfect reflection minimum. The electric field magnitude profile for this optimized thermal emitter has also been plotted in Fig. 4d at the peak emission wavelength, which unequivocally illustrates the enhanced energy density at the location of localized loss element (monolayer graphene). Furthermore, as alluded to previously, the conditions of existence of TIS will be satisfied for only one of the polarization modes; thereby, the thermal emission emerging from the structure would be inherently polarized (see Fig. 3 in Supplementary Information).

Although the optimized design of Fig. 4a can attain near-unity emissivity values, analytical curiosities to obtain perfect emission can also be facilitated. To systematically accomplish this and to gain crucial insights into the operating mechanism, we resort to the theoretical framework of temporal coupled-mode theory (TCMT)^45^, which captures the behavior of our thermal emitter as a leaky mode optical resonator. As per the TCMT framework, a single-mode optical resonator coupled to the outside world through m-ports can be modeled with a symmetric Lorentzian profile with absorption around the resonance frequency ($\omega$), given as

6 $$\begin{aligned} A(\omega )=\frac{4}{m}\left[ \frac{\gamma _{abs}.\gamma _{rad}}{(\omega -\omega _{0})^{2} + (\gamma _{abs} + \gamma _{rad})^{2}} \right] \end{aligned}$$

where $\gamma _{rad}$ is far-field radiative damping rate; $\gamma _{abs}$ is intrinsic loss rate due to ohmic damping mechanisms; *m* is the number of ports.

**Figure 5:**
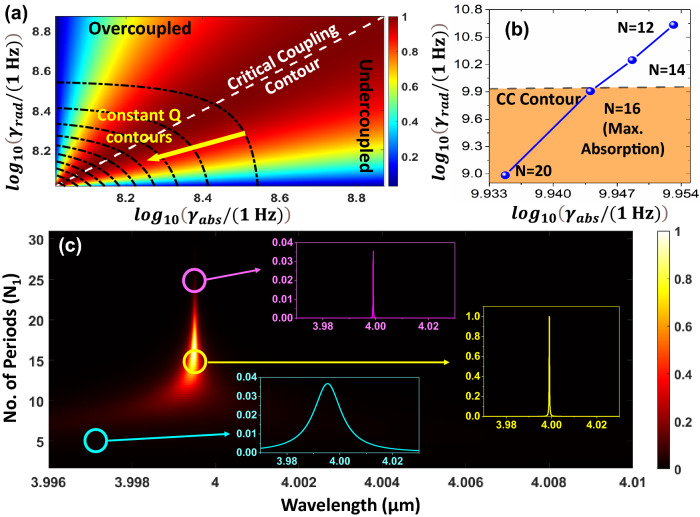
(**a**) TCMT parameter space and notions of absorption optimization for a single mode leaky resonator; (**b**) extraction of perfect absorption operating point in our one-port emitter using TCMT; (**c**) validation of TCMT predictions using TMM and calculated absorption line-profiles.

Whilst the device architecture in our platform suggests the number of ports (*m*) to be two, for attaining perfect absorption we reduce it to single port by blocking transmission . This is accomplished by realizing near-perfect Bragg-reflection from the PhC-II (by setting N$_{2} = 30$), which also prohibits the transmission across the platform. Under such conditions, the absorption in the resonator simplifies to $\frac{\textit{4}\gamma _{rad}.\gamma _{abs}}{\left( \gamma _{rad}+\gamma _{abs}\right) ^{2}}$. Evidently, the key to achieving perfect absorption (efficiency maximization) is to establish a balance between $\gamma _{rad}$ and $\gamma _{abs}$, leading to the critical coupling (CC) of incoming radiation. It is noteworthy that the condition of critical coupling stands independent of the strength of the intrinsic loss channel. Hence, even vanishingly small amounts of loss can lead to perfect absorption. This observation provides an alternative analytical explanation to prior results of near unity emissivity in our platform (for a freestanding monolayer, Graphene absorption is only 0.023). The absorption bandwidth, on the other hand, is dictated by the strength of all damping mechanisms present in the system $(\gamma _{rad}+\gamma _{abs})$. The many implications and predictions of the TCMT framework in a reduced dimension 2D abstract parameter space ($\gamma _{rad}$, $\gamma _{abs}$) have been summed up in Fig. 5a. The order of the associated damping rates has been ascertained by fixing the operating point to the working wavelength of $4~\mu$m. While the efficiency of absorption resonance is dictated by the proximity of an operating point to the CC contour, the resonance bandwidth (and the Q-factor) exhibits a monotonically decreasing (increasing) behavior along the marked direction. The contiguous parameter space is then searched for a local maximum with the corresponding running variable in the device to be the number of periods (N$_{1}$) in PhC-I. The mapping of variation in N$_{1}$ to $\gamma _{rad}$ and $\gamma _{abs}$ has been done by fitting the TCMT responses to the TMM calculations, and the results are presented in Fig. 5b. Starting from N$_{1} = 12$, as we increase N$_{1}$, the operating point moves from overcoupled to undercoupled regime, crossing the critical coupling contour at N$_{1} = 16$, which marks the TCMT prediction for perfect absorption. An exhaustive parameter search of Fig. 5c performed solely based on TMM analysis provides testimony to such claims.

**Figure 6:**
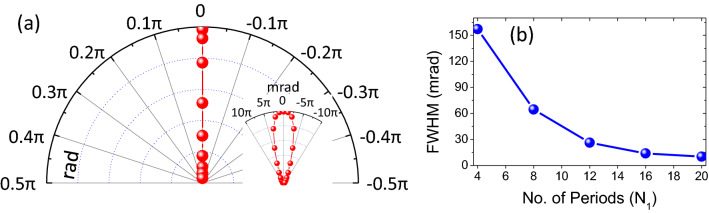
(**a**) Emissivity patterns in polar coordinates at the operating wavelength for the proposed thermal emitter; (**b**) variation of the emission lobe beamwidth with N$_{1}$.

From spectral characteristics, we now move on to the spatial characteristics of the designed thermal emitter. It is noteworthy to ponder upon the directional emission characteristics of the proposed thermal emitter, which is ascertained from the angle-resolved emissivity pattern at the operating wavelength, as depicted in Fig. 6a. From these patterns, the angular width of the radiation lobe comes out to be 27 mrad, establishing its ultra-narrow angular emission behavior. In Fig. 6b, we plot the angular width of the emission as a function of N$_{1}$. It depicts that the FWHM of the emission lobe exhibits a monotonically decreasing behavior with an increase in the number of periods, and hence, the directional emission characteristics can further be improved by incorporating more periods in the platform. The value of peak emission, though, does not exhibit such a monotonic behavior and, in line with the predictions of the TCMT framework, starts to fall off beyond an optimum point.

Lastly, we mention some design considerations from the viewpoint of fabrication conduciveness. Firstly, the structure in Fig. 4a can be simplified to some extent by replacing the monolayer graphene sheet with an absorbing film. Such a design would entail a compromise in thermal emission performance owing to the relatively distributed nature of the loss element, but can be instrumental in situations demanding fabrication simplifications. Although the non-negligible thickness and the resulting modifications brought about by a thin absorbing film do not hamper the existence of TIS; however, as established by the TCMT framework, the critical coupling condition would be altered, leading to degradation in spectral emission characteristics. Specifics and the calculated performance for one such design incorporating thin plasmonic metal film (of gold) are provided in Fig. 4 in Supplementary Information. Therefore, although graphene is used only as a loss element in our structure and has no role to play regarding the qualitative response of TIS, its incorporation becomes critical for obtaining high quality factors.

An even greater fabrication simplification is possible by resorting to a corresponding in-plane configuration, as provided in^46^. The nanobeam PhC configuration discussed in this work deviates from the vertical stacking of layers; instead, the constituent equivalent layers are stacked in-plane with a corresponding change in the light propagation direction.

In conclusion, we have designed and analyzed a quasi-monochromatic and highly directional thermal emitter by harnessing a topological interface state. The TIS owes its existence to the EO-induced small perturbations in the LiNbO$_{3}$ RI and the associated modalities of topological phase transitions, which being free from carrier transport mechanisms and heat diffusion dynamics can facilitate ultrahigh-speed mid-IR signal processing. Our approach of capitalizing on a defect-less and lithography-free geometric phase-based architecture offers advantages of volume production and ensures structural stability, opening the prospects for the unobtrusive development of mid-IR integrated photonics hot thermal emitters.

## Supplementary Information

Supplementary Information.
