# Supplementary material for: Realizing quasi-monochromatic switchable thermal emission from electro-optically induced topological phase transitions
Source: Sci Rep. 2022 May 5;12:7400. doi: 10.1038/s41598-022-11410-6 (PMC9072548; doi:10.1038/s41598-022-11410-6)
Supplement: Supplementary file 1 — Supplementary Information. [file 41598_2022_11410_MOESM1_ESM.pdf]

Supplementary Information

Realizing Quasi-monochromatic Switchable Thermal Emission from Electro-Optically induced Topological Phase Transitions

I. ELECTRO OPTIC COEFFICIENT TENSOR FOR LINBO$_3$

In this work, we have utilized the linear electro-optic (EO) effect of $LiNbO_3$ to realize an electrically switchable TIS modulator. The change of RI, in response to the applied electric field, is given by the following relation,

$$\Delta(\frac{1}{n^2})_{ij} = \sum_k r_{ijk} E_k, \tag{1}$$

where $\Delta(\frac{1}{n^2})_{ij}$ is the second-rank tensor of the change in relative permittivity, r_{ijk} is a third-rank linear EO coefficient tensor, E_k is electric field component along k, and i,j,k are x,y, and z respectively Weis and Gaylord (1985); Yariv and Yeh (1984). The reduced EO coefficient tensor of $LiNbO_3$ is given by,

$$r_{ijk} = \begin{bmatrix} 0 & -r_{22} & r_{13} \\ 0 & r_{22} & r_{13} \\ 0 & 0 & r_{33} \\ 0 & r_{42} & 0 \\ r_{42} & 0 & 0 \\ -r_{22} & 0 & 0 \end{bmatrix}, \tag{2}$$

where $r_{22} = 3.1pm/V$, $r_{13} = 6.5pm/V$, $r_{33} = 28pm/V$, and $r_{42} = 23pm/V$ (for the mid-IR wavelengths), are the four independent coefficients, which describe the linear EO effect in $LiNbO_3$ Jazbinšek and Zgonik (2002); Turner (1966); Shuto and Amano (1995); Roussey *et al.* (2006). Among all, r_{33} is the largest linear EO (Pockel's) coefficient. Therefore, we choose a configuration of Figure 3(a) such that the input polarization is along the optic axis direction (z-direction) and r_{33} is employed.

II. TOPOLOGICAL INTERFACE STATE MODULATOR

For the purpose of comparative evaluation, the calculated performances of TIS modulator in ON-state and OFF-state are given in FIG-1.

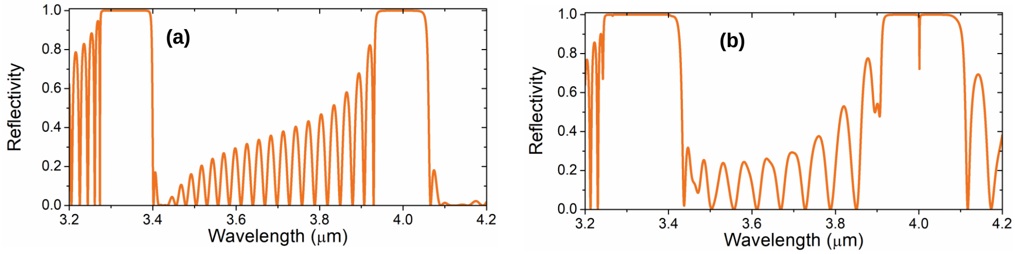

FIG. 1. Calculated spectral performance of TIS modulator in (a) OFF-sate; (b) ON-state.

III. ELECTRIC FIELD PROFILE FOR TIS

The electric field magnitude profile corresponding to the TIS obtained in Figure.3 of the main manuscript is plotted in FIG-2, at the resonance wavelength of $4\mu m$. The expo-

nentially decaying nature of field values, away from the interface, confirm its surface state nature.

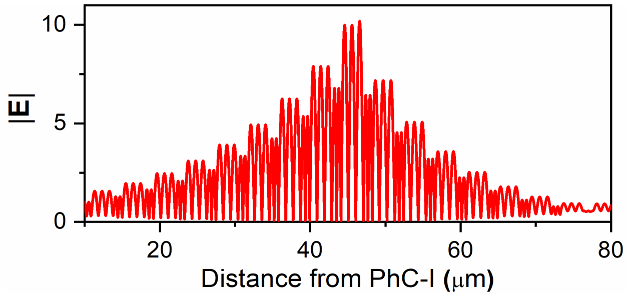

FIG. 2. Calculated spectral performance of switchable thermal emitter with y-polarization for (a) ON-state; (b) OFF-state.

IV. GRAPHENE CONDUCTIVITY AND OPTICAL CONSTANTS

Graphene conductivity can be described consisting of two contributions:

$$\sigma = \sigma_{inter} + \sigma_{intra}, \tag{3}$$

$$\sigma_{inter} = \frac{ie^2}{4\pi\hbar} ln \left[\frac{2E_F - (\omega + i\xi^{-1})\hbar}{2E_F + (\omega + i\xi^{-1})\hbar} \right], \tag{4}$$

$$\sigma_{intra} = \frac{ie^2 K_B T}{\pi\hbar^2(\omega + i\xi^{-1})} \left[\frac{E_F}{K_B T} + 2ln(e^{\frac{-E_F}{K_B T}} + 1) \right], \tag{5}$$

where E_F is the Fermi level, τ is the relaxation rate and rest of the parameters have standard meaning as defined in Ref [31] of the main manuscript. Using this conductivity value the relative dielectric permittivity for the graphene layer can be ascertained as

$$\epsilon_{graphene} = 1 + \frac{i\sigma}{\omega\epsilon_0 d_g}. \tag{6}$$

The relative permeability of graphene has been taken to be unity in all the calculations.

V. POLARIZATION PERFORMANCE OF THE SWITCHABLE THERMAL EMITTER

The proposed thermal emitter exhibits an inherent polarization discrimination, the genesis of which can be explained as follows:

In the employed configuration of Fig. 4(a) of main manuscript, the optic axis and the applied voltage are along the z-axis, while the light is propagating along the x-axis. Such a configuration allows us to use the maximum Pockel's coefficient, and the corresponding maximum change of refractive index is along the z-axis. It is given by the following formula,

$$\delta n_z = -\frac{n_e^3}{2} r_{33} E_z, \tag{7}$$

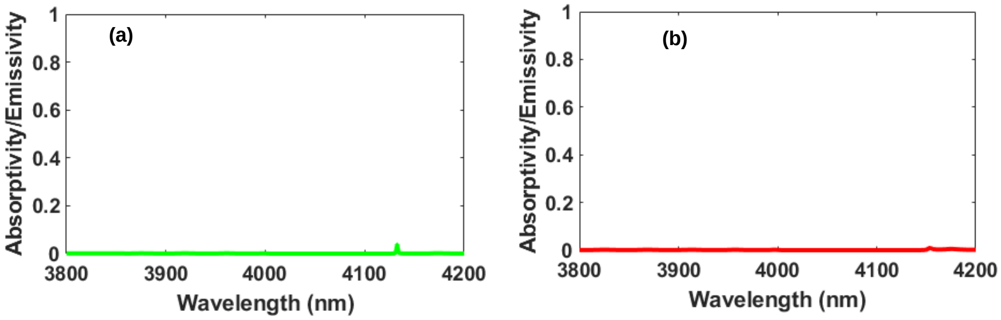

FIG. 3. Calculated spectral performance of switchable thermal emitter with y-polarization for (a) ON-state; (b) OFF-state.

where $n_e = 2.04$ is extraordinary RI and $r_{33} = 28pm/V$. Furthermore, the applied voltage along the z-axis provides the RI change in the n_y as well, which is determined by the following formula,

$$\delta n_y = -\frac{n_o^3}{2} r_{13} E_z, \tag{8}$$

where $n_o = 2.11$ is ordinary RI and $r_{13} = 6.5pm/V$.

Since the r_{13} is roughly five times smaller in value than r_{33}, our platform works as intended for the z-polarized light only. Thus, the emitted thermal radiation will inherently be polarized along z-axis. These conclusions are further substantiated by the calculations done for the y-polarization, as depicted in FIG-3.

VI. ALTERNATE DESIGN FOR FABRICATION SIMPLIFICATION

As mentioned in the main manuscript, we provide here an alternate design for the switchable thermal emitter which incorporates a thin gold layer (of 3nm thickness), in place of the monolayer graphene. The optical constants of gold film are modeled using the Drude formula

$$\epsilon(\omega) = 1 - \frac{\omega_p^2}{\omega(\omega + i\gamma)} \tag{9}$$

where $\omega_p = 1.3677 \times 10^4$ THz and $\gamma = 6.5$ THz) Ordal *et al.* (1983).

The optimization procedure, as described in the main manuscript, has been repeated for this alternate design as well. The framework provides us an optimum number of periods for this design to be N_1=8 and N_2=10. The corresponding calculated spectral performance has been provided in FIG-4.

VII. AN ALTERNATE SIMPLIFIED DESCRIPTION FOR THE GENESIS OF THE TOPOLOGCAL INTERFACE STATE

Since the reflection phase of our topological PhCs is non-trivial, we provide here an alternate description for qualitatively looking at the observed phenomena. To this objective, we resort to one of our recent works on metasurface-based cavities Gupta *et al.* (2022).

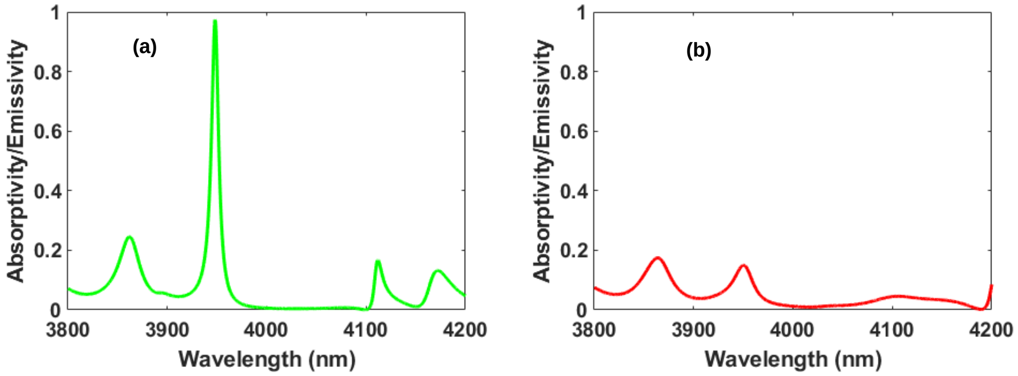

FIG. 4. Calculated spectral performance for an alternate design of switchable thermal emitter (incorporating thin gold film) for (a) ON-state; (b) OFF-state.

where we have experimentally demonstrated a metasurface-based absorber at microwave frequencies and presented a highly simplified and intuitive picture for ascertaining the scattering characteristics of such structured photonic cavities. Simply put, metasurfaces assign a non-trivial phase-space to the conventional Fabry-Perot cavities. Following the partial wave summation, we obtain a general expression for the reflection coefficient:

$$\tilde{r}_{total} = |\tilde{r}_{12}|exp^{\psi_{12}} + [|\tilde{t}_{12}|exp^{\zeta_{12}}|\tilde{t}_{21}|exp^{\zeta_{21}}|\tilde{r}_{23}|exp^{\psi_{23}}\frac{e^{i2kl_{\text{cavity}}}}{(1-|\tilde{r}_{21}|exp^{\psi_{21}}|\tilde{r}_{23}|exp^{\psi_{23}}e^{i2kl_{\text{cavity}}}})] \tag{10}$$

where, k is the propagation constant inside the cavity, and $|\tilde{r}_{ij}|$, $|\tilde{t}_{ij}|$ are the reflection/transmission coefficients of the partial waves generated at the interfaces while going from medium i to j, ψ_{23} and ζ_{ij} are the phases associated with partial wave reflection and transmission coefficients.

With the help of this expression, we can readily observe that even in the case of vanishing cavity lengths, the possibility of resonance can be sustained. Specifically, based on the self-consistency constraints, we arrive at the resonance condition:

$$e^{i2kl_{\text{cavity}}+\psi_{23}+\psi_{21}} = 1 \tag{11}$$

which, for a non-trivial phase response, can be satisfied even for $l_{\text{cavity}} \to 0$. This intuitive picture enables us to qualitatively appreciate the possibility of resonance in a vanishing thickness cavity. The above-mentioned analysis can be well utilized (although with the risk of an over-simplified perspective) in our present work to underline some of the novel features of topologically non-trivial PhCs (here, we will be calling them topological reflectors). As the phase response of these reflectors is also non-trivial (Figure 3(b) in the main manuscript), it promulgates the possibility of resonance for vanishing cavity thicknesses (as characterized by equation (11) presented above). Its realization would require near opposite phase characteristics for the two PhCs and that's what has been obtained in Figure 3(b) of main manuscript.

REFERENCES

Gupta, N. K., Singh, G., Wanare, H., Ramakrishna, S. A., Srivastava, K. V., and Ramkumar, J., "A low-profile consolidated metastructure for multispectral signature management," Journal of Optics (2022).

Jazbinšek, M.and Zgonik, M., "Material tensor parameters of linbo3 relevant for electro-and elasto-optics," Applied Physics B **74**, 407–414 (2002).

Ordal, M. A., Long, L., Bell, R., Bell, S., Bell, R., Alexander, R., and Ward, C., "Optical properties of the metals al, co, cu, au, fe, pb, ni, pd, pt, ag, ti, and w in the infrared and far infrared," Applied Optics **22**, 1099–1119 (1983).

Roussey, M., Bernal, M.-P., Courjal, N., Van Labeke, D., Baida, F., and Salut, R., "Electro-optic effect exaltation on lithium niobate photonic crystals due to slow photons," Applied Physics Letters **89**, 241110 (2006).

Shuto, Y.and Amano, M., "Reflection measurement technique of electro-optic coefficients in lithium niobate crystals and poled polymer films," Journal of Applied Physics **77**, 4632–4638 (1995).

Turner, E., "High-frequency electro-optic coefficients of lithium niobate," Applied Physics Letters **8**, 303–304 (1966).

Weis, R.and Gaylord, T., "Lithium niobate: summary of physical properties and crystal structure," Applied Physics A **37**, 191–203 (1985).

Yariv, A.and Yeh, P., *Optical waves in crystals*, Vol. 5 (Wiley New York, 1984).
